# *Candida albicans *versus *Mycobacterium tuberculosis*: infection-specific human immune responses

**DOI:** 10.1186/cc14029

**Published:** 2014-12-03

**Authors:** JPLF Saraiva, R König

**Affiliations:** 1 Network Modelling, Hans-Knöll-Institut, Jena, Germany; 2 Centre for Sepsis Control and Care, Jena University Hospital, Jena, Germany

## Introduction

Systemic infection of the human host can arise from several pathogenic bacteria as well as from fungal species, and it is of high clinical relevance to trace back the nature of infection from the host response. Amongst these systemic infections are sepsis inducers *Candida albicans *and *Mycobacterium tuberculosis*, responsible for a great amount of worldwide deaths [1]. Sepsis, however, does not necessarily originate from infection. Traumas and non-infection-based injuries can also trigger an unbound inflammatory response from the human host most commonly known as systemic inflammatory response syndrome. Both cases, infection and non-infection, nevertheless display similar clinical symptoms with significantly better recovery times of patients included in the latter. Despite the clinical similarities, studies have suggested that distinct infection-induced host responses from different pathogens occur, namely at the signalling pathway level [2].

## Methods

Studies have been performed focused on common gene expression profiles between several pathogens [3]. However, understanding the difference in immune responses between these two pathogens, but not exclusively, might lead to better diagnostic tools and treatment decision-making. Relying on systems biology concepts and bioinformatics tools, we use gene expression data to distinguish *C. albicans *and *M. tuberculosis *infections in the human host; for example, cellular regulation and communication between host immune cells and pathogens [4,5].

## Results

We have, in a first analysis, focused on the cytokine-cytokine signalling pathway due to its role in inflammation response towards infections. Genes belonging to the IL-2 cytokine family are only expressed when facing infection by *C. albicans *in comparison to *M. tuberculosis *suggesting a tendency towards B-cell proliferation and production of antibodies. However, during infection caused by *M. tuberculosis*, no significant changes in gene expression occur in this pathway that indicates a specific immune response for this pathogen. Further analysis of additional signalling pathways might highlight other human infection-specific immune responses in regards to the pathogens considered.

## Conclusion

Next we will focus on developing a mathematical model capable of simulating such immune responses and possibly identifying genes and pathways which might clarify how these inflammation responses can be targeted, countered and moderated [6].

## References

[B1] Angus Epidemiology of severe sepsis in the United States: analysis of incidence, outcome, and associated costs of care Crit Care Med 2001 29 1303 10.1097/00003246-200107000-00002 11445675

[B2] Smeekens SP Functional genomics identifies type I interferon pathway as central for host defense against Candida albicans Nat Commun 2013 4 1342 23299892 10.1038/ncomms2343 PMC3625375

[B3] Jenner RG Young RA Insights into host responses against pathogens from transcriptional profiling Nat Rev Microbiol 2005 3 281 294 10.1038/nrmicro1126 15806094

[B4] Väremo L Nielsen J Nookaew I Enriching the gene set analysis of genome-wide data by incorporating directionality of gene expression and combining statistical hypotheses and methods Nucleic Acids Res 2013 41 4378 4391 10.1093/nar/gkt111 23444143 PMC3632109

[B5] Luo W Brouwer C Pathview: an R/Bioconductor package for pathway-based data integration and visualization Bioinformatics 2013 29 1830 1831 10.1093/bioinformatics/btt285 23740750 PMC3702256

[B6] Mogensen TH Pathogen recognition and inflammatory signaling in innate immune defenses Clin Microbiol Rev 2009 22 240 273 10.1128/CMR.00046-08 19366914 PMC2668232

